# Supplementary material for: Detailed molecular and epigenetic characterization of the pig IPEC-J2 and chicken SL-29 cell lines
Source: iScience. 2023 Feb 20;26(3):106252. doi: 10.1016/j.isci.2023.106252 (PMC10018572; doi:10.1016/j.isci.2023.106252)
Supplement: Data S2. Complete homer output for identified motifs in Chicken SL-29, related to Tables 5 and 6 — Homer motif analysis results for histone modifications H3K4me1, H3K4me3, H3K27ac, enhancers, and ATAC-seq of chicken SL-29 cell line. Parameters for possible false positives is as mentioned earlier for S5. [file mmc3.zip › Data_S2/S6/Chicken_SL_29/peak_files_enhancers/homerResults/motif31.similar.html]

motif31

## Information for motif31

A
C
T
G
A
G
T
C
A
C
T
G
C
G
T
A
C
G
T
A
A
C
G
T
C
G
A
T
A
C
T
G
C
T
A
G
A
C
T
G
  
Reverse Opposite:  

G
T
A
C
A
G
T
C
A
G
T
C
C
G
T
A
C
G
T
A
A
C
G
T
A
C
G
T
A
G
T
C
C
T
A
G
G
T
A
C
  

|  |  |
| --- | --- |
| p-value: | 1e-10 |
| log p-value: | -2.327e+01 |
| Information Content per bp: | 1.866 |
| Number of Target Sequences with motif | 16.0 |
| Percentage of Target Sequences with motif | 0.09% |
| Number of Background Sequences with motif | 3.8 |
| Percentage of Background Sequences with motif | 0.01% |
| Average Position of motif in Targets | 172.0 +/- 78.6bp |
| Average Position of motif in Background | 176.0 +/- 60.1bp |
| Strand Bias (log2 ratio + to - strand density) | -1.0 |
| Multiplicity (# of sites on avg that occur together) | 1.31 |
| Motif File: | file (matrix) reverse opposite |

### Similar de novo motifs found

|  |  |  |  |  |  |  |  |
| --- | --- | --- | --- | --- | --- | --- | --- |
| Rank | Match Score | Redundant Motif | P-value | log P-value | % of Targets | % of Background | Motif file |
